# Photoplethysmography and Deep Learning: Enhancing Hypertension Risk Stratification

**DOI:** 10.3390/bios8040101

**Published:** 2018-10-26

**Authors:** Yongbo Liang, Zhencheng Chen, Rabab Ward, Mohamed Elgendi

**Affiliations:** 1 School of Electrical Engineering, Guilin University of Electronic Technology, Guilin 541004, China; liangyongbo001@gmail.com (Y.L.); chenzhcheng@163.com (Z.C.); 2 School of Electrical and Computer Engineering, University of British Columbia, BC V6T 1Z4, Canada; rababw@ece.ubc.ca; 3 Faculty of Medicine, University of British Columbia, BC V1Y 1T3, Canada; 4 BC Children’s and Women’s Hospital, Vancouver, BC V6H 3N1, Canada

**Keywords:** pulse morphology, pulse oximeter, blood pressure monitoring, pulse arrival time, global health, digital medicine, wearable devices, hypertension assessment, hypertension evaluation

## Abstract

Blood pressure is a basic physiological parameter in the cardiovascular circulatory system. Long-term abnormal blood pressure will lead to various cardiovascular diseases, making the early detection and assessment of hypertension profoundly significant for the prevention and treatment of cardiovascular diseases. In this paper, we investigate whether or not deep learning can provide better results for hypertension risk stratification when compared to the classical signal processing and feature extraction methods. We tested a deep learning method for the classification and evaluation of hypertension using photoplethysmography (PPG) signals based on the continuous wavelet transform (using Morse) and pretrained convolutional neural network (using GoogLeNet). We collected 121 data recordings from the Multiparameter Intelligent Monitoring in Intensive Care (MIMIC) Database, each containing arterial blood pressure (ABP) and photoplethysmography (PPG) signals. The ABP signals were utilized to extract blood pressure category labels, and the PPG signals were used to train and test the model. According to the seventh report of the Joint National Committee, blood pressure levels are categorized as normotension (NT), prehypertension (PHT), and hypertension (HT). For the early diagnosis and assessment of HT, the timely detection of PHT and the accurate diagnosis of HT are significant. Therefore, three HT classification trials were set: NT vs. PHT, NT vs. HT, and (NT + PHT) vs. HT. The F-scores of these three classification trials were 80.52%, 92.55%, and 82.95%, respectively. The tested deep method achieved higher accuracy for hypertension risk stratification when compared to the classical signal processing and feature extraction method. Additionally, the method achieved comparable results to another approach that requires electrocardiogram and PPG signals.

## 1. Introduction

Cardiovascular diseases (CVDs) have become a major contributor to human mortality [1]. According to the latest statistics produced by the World Health Organization (WHO), the mortality rate from CVDs will rise from 246 people per one million in 2015 to 264 people per one million in 2030 [2,3]. It is known that abnormal blood pressure can produce many complications for the heart, kidneys, and other vital organs, causing irreversible injury. Therefore, early diagnosis, treatment, and control of hypertension could play an important role in the prevention and treatment of CVDs. However, due to the complexity of the formation of hypertension, there is still no effective means to completely cure it; only constant blood pressure measurement and hypertension assessment, which enable adjustments to the drug situation, allow the stabilization of blood pressure at healthy levels [4,5]. A more invasive blood pressure measurement method, which is the gold standard, involves inserting a catheter into an artery to conduct real-time monitoring. This approach is only suitable for critical patients and also carries the risk of infection. The most common technique used for blood pressure measurement is Korotkoff’s sound and oscillographic method [6]. Both of these techniques require the use of an upper arm cuff and utilize the cuff pressure and release process to detect systolic and diastolic pressure. These two intelligent methods are the most widely used and are continuously being optimized. Their simple operation and reliable results have been accepted by medical staff and patients. Although this technology is widely accepted, the requirement of a cuff creates obstacles for its wider spread and application.

In recent years, new methods of blood pressure detection and evaluation have been proposed; the methods based on arterial wave propagation theory [7,8,9,10] and photoplethysmogram (PPG) morphology theory [11,12,13] have been studied most. The former method assesses blood pressure levels by measuring pulse transit time (PTT) [14]. This method usually requires two physiological signals, such as electrocardiogram (ECG) and PPG signals [15]. This approach has been explored by a number of past studies, verifying the feasibility of the solution. The acquisition of two signals increases the stability of the method; however, it increases the operational complexity, especially the acquisition of the ECG. It does not require the sleeve, but there are still more problems to be solved. The latter method assesses blood pressure levels by establishing a PPG morphological feature model [16]. This method requires a PPG signal of higher quality, such as high sampling rates and sampling precision, and is very sensitive to many kinds of noises; this also limits its wide application.

At present, health care has entered a new era. Deep learning has become a powerful computational method that has been used to solve many types of identification and classification problems. Deep learning is used to automatically learn the optimal features of raw signals. The typical framework of deep learning includes deep belief networks (DBN), stacked autoencoders (SAE), convolutional neural networks (CNN), etc. [17]. Deep learning technology has rapidly developed in recent years and has been applied, for example, to image recognition [18], speech recognition, and object identification [19]. In the field of biomedical engineering, a large amount of medical data is also being recognized and mined. Some researchers have used this powerful method for practical medical applications, such as breast cancer detection, heartbeat classification using ECG signals, brain tumor image recognition, etc. [20,21,22]. With the huge amount of medical data, deep learning also has the potential to play a more important role in mining health information from wearables, monitoring medical information, and diagnosing disease, among other fields. 

It is known that human tissues and organs undergo certain effects and experience changes during different stages of hypertension. This is especially true for the cardiovascular circulatory system. PPG signals contain abundant physiological information on cardiovascular circulatory processing. A series of blood-pressure-level assessments based on ECG and PPG signals were recently performed by the authors. This recent work [23] showed that the replacement of arterial blood pressure (ABP) with PPG signals is very promising. Another recent paper [24] by our group, using the same dataset as in this publication, compared the pulse arrival time (PAT, based on ECG and PPG signals) and PPG morphological theory (based only on PPG). The PAT feature and many PPG morphological features were extracted and used to classify blood pressure (BP) categories individually and in combination. The limitations of using feature extraction are (1) the feature points of each heartbeat needed to be extracted correctly [25,26,27], (2) the ECG and PPG signals have to be of high quality, and (3) optimal filtering is required [28,29,30,31].

To eliminate the classical feature extraction phase, we introduced a transfer learning method based on the pretrained convolutional neural network (CNN). Common pretrained CNN methods include AlexNet, VGGNet, and GoogLeNet, which are trained based on the ImageNet Large Scale Visual Recognition Challenge (ILSVRC) dataset [2]. In this paper, we propose a method of classifying hypertension using continuous wavelet transformation and GoogLeNet [32], which only needs the PPG signal to realize the identification and classification of hypertension. It has many advantages, such as having a low demand for the original signal, being rapid, and having a wide range of applications. 

## 2. Materials and Methods

### 2.1. Data Acquisition

In this study, the ABP (measured using a catheter in the radial artery) and photoplethysmograph (PPG) signals were used to carry out the assessment and classification of hypertension. They were collected from the Multiparameter Intelligent Monitoring in Intensive Care Database (MIMIC [33,34]), which is a free-to-use database that contains multiple and complex-parameter recordings of tens of thousands of intensive care unit (ICU) patients. The ABP signals were used to extract the blood pressure level labels, which are defined as normotension (NT), prehypertension (PHT), and hypertension (HT) in the seventh report of the Joint National Committee on Prevention, Detection, Evaluation, and Treatment of High Blood Pressure (JNC7 [35]) by the US National Institutes of Health. Therefore, the records with missing peaks, pulsus bisferiens, no signal (sensor-off), and so on were excluded from this study to extract the blood pressure labels accurately. Ultimately, 121 subjects’ recordings were collected, each with stable, complete ABP and PPG signals and with 120 s lengths and 125 Hz sampling rates. 

### 2.2. Signal Pre-Processing

**Figure 1:**
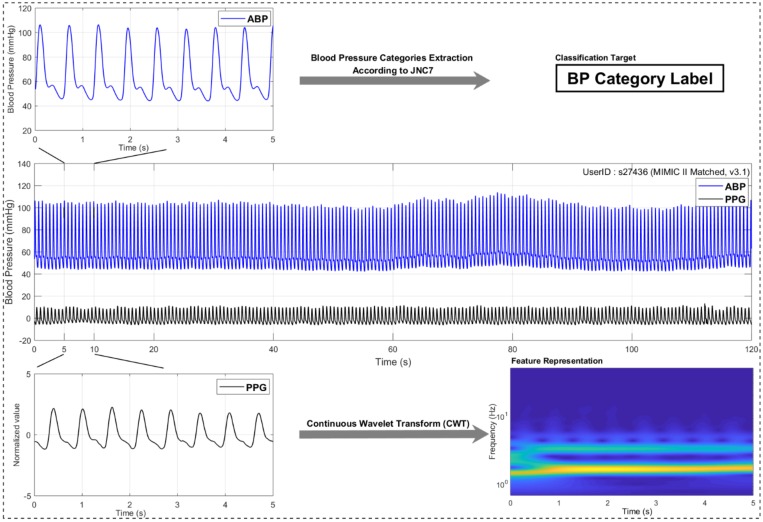
A signal processing structure. Note: PPG stands for *photoplethysmogram*, ECG stands for *electrocardiogram*, BP stands for *blood pressure*, and JNC7 stands for the Seventh Report of the Joint National Committee.

Each recording consisted of two signals. These were ABP and PPG signals, which were used as the target source and predicted source, respectively. The ABP signals were not processed and were only used to extract systolic blood pressure (SBP) and diastolic blood pressure (DBP) and then to determine the blood pressure level labels as described in the JNC7. The PPG signals were processed with a 0.5–10 Hz Chebyshev II bandpass filter to remove the noise [36]. Following this, amplitude normalization was performed on the filtered PPG signals. In addition, the ABP and processed PPG signals were cut into 5 s signal segments. Figure 1 shows the structure of the signal processing.

### 2.3. PPG Signal Transformation Using Continuous Wavelet Transform (CWT)

Since the pretrained CNN network accepts Red-Green-Blue (RGB) images as input, the raw PPG signals of this paper were one-dimensional vector signals. Therefore, the raw PPG signals were first converted to produce a feature representation. Following this, we adopted the continuous wavelet transform (CWT) to convert the PPG signals to RGB images. CWT has been considered an effective method for analyzing frequency information along with time. Each PPG signal segment was converted to a time-frequency representation, called a scalogram, by continuous wavelet transform.

**Figure 2:**
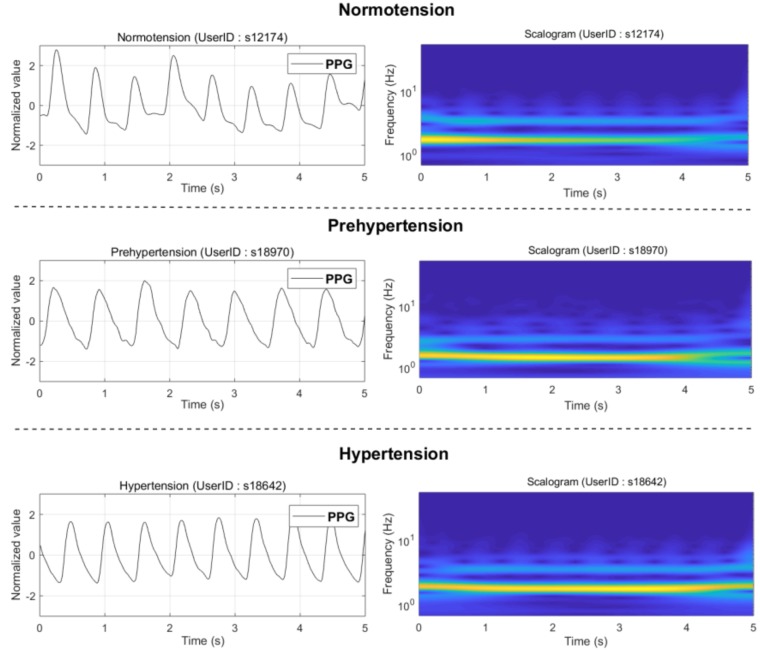
The scalogram cases of the three different blood pressure categories. Note: PPG stands for *photoplethysmogram*.

A scalogram, which is plotted as a graph of time and frequency, is the absolute value of the continuous wavelet transform coefficients of a signal. Compared with a spectrogram, a scalogram can better identify the low-frequency or fast-changing frequency component of the signal. PPG signals are a fusion of heart activity, vascular relaxation processes, and microcirculation system status; therefore, its time–frequency domain information is rich and diverse. The PPG signal is transformed into a scalogram using the wavelet coefficients of continuous wavelet transform, which can be used to locate different frequency components. This recognizable scale RGB image is very beneficial for extracting and learning about the features of convolutional neural networks, especially pretrained CNN. In this study, we obtained the absolute value of the wavelet coefficients for each signal segment using analytic *morse* (*3,60*) wavelet, setting the *VoicesperOctave* to 12 through the *cwtfilterbank* of the Wavelet toolbox, and then converting them to RGB images. Finally, the RGB images were adjusted to the size of 224 × 224 × 3, which is a requirement of GoogLeNet. Following this, the image-like data were fed into the pretrained CNN to produce their corresponding CNN features. Figure 2 illustrates the scalograms of the three different blood pressure categories.

### 2.4. Pretrained Convolutional Neural Network (GoogLeNet)

GoogLeNet is a pretrained convolutional neural network structure proposed by Szegedy in 2015 [37]. It uses the inception structure to build a deep learning neural network. This structure makes good use of the computational resources in the network without increasing the computational load or increasing the width and depth of the network. Due to GoogLeNet’s ability to generalize extracted depth characteristics, it can be applied to a variety of computer vision identification and classification problems. GoogLeNet has 22 layers and is trained on 1.2 million high-resolution RGB images from the ImageNet dataset. It can classify these images into 1000 different categories with state-of-the-art performance. The categories mainly include conventional things, such as cats, dogs, fruits, cars, etc. GoogLeNet is also regarded as a trained deep learning network that can be converted to new image classification applications using a smaller number of training images, such as medical image diagnosis, signal spectrum recognition, etc. In previous studies, some researchers have also shown that the classification of biosignals into categories can be achieved using GoogLeNet [38,39]. 

Transfer learning is one structural model of deep learning [40]. In brief, one basic convolutional neural network can be trained based on a large database. Some successful pretrained CNNs include LeNet, AlexNet, VGG, and GoogLeNet. These pretrained CNNs can be directly used to recognize more than 1000 objects. As the generalization ability of the extracted depth characteristics by transfer learning is very strong, it can be applied to other, different computer vision identification and classification problems (e.g., MRI medical images and physiological signals). The retraining does not change the structure; rather, it fine-tunes the parameters. With regard to RGB images, different images have varying features, such as edges, texture, etc. Thus, transfer learning can have wider applications if it can be transformed to RGB images.

For this paper, the signal classification study was transferred to image identification and classification by continuous wavelet transformation. GoogLeNet, which is fine-tuned and trained based on the dataset in this study, solved the problem of signal classification effectively. Compared with AlexNet and VGG networks, which adopt convolutional layers and full-connection layers [32], GoogLeNet has deeper depth but a smaller model size [37]. It adopts sub-network and inception for the first time and shows superior performance. Deeper network depth and smaller model size have become the main reasons for selecting a pretrained CNN network.

GoogLeNet adopts the 224 × 224 × 3 sized RGB image as the input. However, the signals of this study were discrete sampling signals with sampling rates of 125 Hz and 5 s in length. Therefore, all training and testing signals were first processed by continuous wavelet transform and then converted to 224 × 224 × 3 sized RGB images. Each image represented an independent signal segment. In this study, the training of GoogLeNet used the Batch and Epoch settings; the minimum Batch size was 15, while the maximum Epoch was 20. In addition, Fully Connected was set as “loss3-classifier”, and Softmax was set as “prob.”

### 2.5. Hypertension Classification

**Figure 3:**
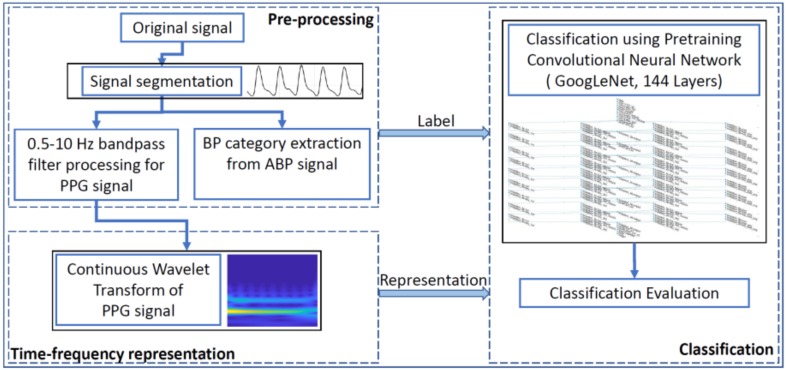
Flowchart of the study.

As we know, according to the hypertension categories of the JNC7 report, blood pressure levels for adults are divided into normotension (NT), prehypertension (PHT), and hypertension (HT). In order to classify hypertension based on a PPG signal, three classification experiments were conducted: NT versus PHT, NT versus HT, and non-HT (NT + PHT) versus HT. This study used 80% of the dataset for training, and the remaining 20% was used for testing. All the signal processing, modeling, and evaluating were carried out in MATLAB software (R2018a version), which is developed and released by the MathWorks company. In addition, the training and testing of the model required the Neural Network Toolbox Model for the GoogLeNet Network support package. Refer to Figure 3 for the flowchart of this study. 

## 3. Results

In this study, data recordings with a sampling rate of 125 Hz containing ABP and PPG signals were collected from the MIMIC physiological database. Raw ABP signals, which were used for the extraction of blood pressure categories, were not processed. Raw PPG signals were processed with a 0.5–10 Hz Chebyshev II bandpass filter [36] and then processed by normalization. Next, each recording was cut into several 5 s signal segments. Each PPG signal segment was converted into a scalogram by continuous wavelet transform and then fed into the training and testing of the GoogLeNet model. Sensitivity, specificity, accuracy, and F1 scores were used to evaluate the classification performance, which was calculated based on the classification confusion matrix. 

**Figure 4:**
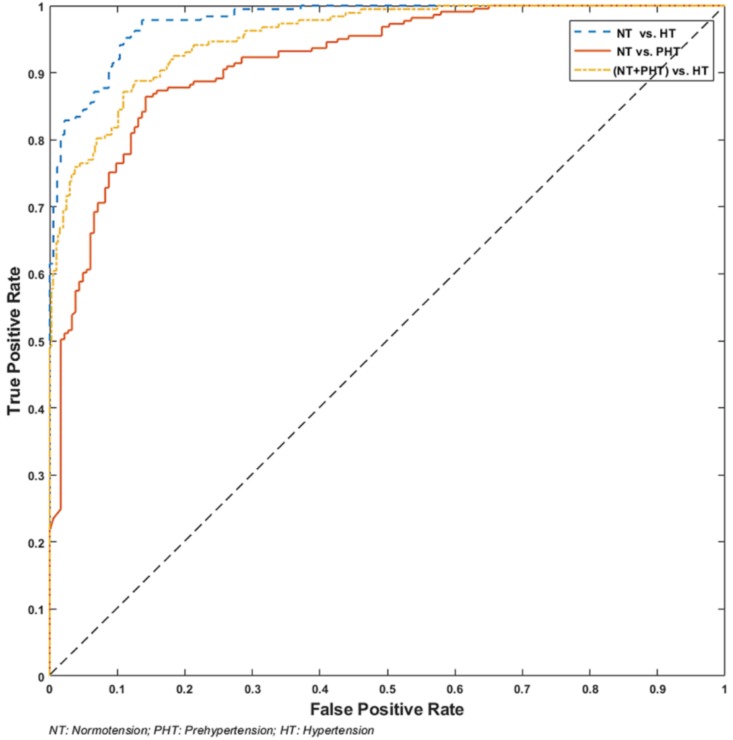
The receiver operating characteristic (ROC) curve of the three classification trials.

Table 1 shows the classification performance of the three trials. Figure 4 illustrates the receiver operating characteristic (ROC) curve of the three classification trials. To our knowledge, there have not been any comparable studies addressing this research context. Artery wave propagation theory, which uses ECG and PPG signals, and PPG morphological theory, which uses PPG signals, has been most commonly studied [41]. This study used raw PPG signals and did not extract morphological features to classify the BP into categories. Therefore, in order to evaluate the performance of this study, a comparative analysis with the artery wave propagation theory and PPG morphological theory were conducted on the same dataset, as shown in Table 1.

Note that there was no overfitting problem in this study. We compared the classification accuracy based on the training set and testing set. The accuracy based on the testing set was similar to the accuracy based on the training set. This meant that the model was stable for predictions when new data were used.

In previous research [24], blood pressure classification using PAT and PPG features extracted from ECG and PPG signals was also conducted using the same dataset in this study. Table 1 shows that the F1 scores found in this study were higher than the classifier using 10 PPG features. This indicates that GoogLeNet achieves better performance than extracting PPG morphological features based on poor quality PPG signals collected from elderly ICU patients. In addition, this study achieved a performance similar to artery wave propagation theory. This indicates that this study, which exclusively used raw PPG signals, has promising potential to replace the ECG and PPG hypertension classification method.

## 4. Discussion

The conventional evaluation of hypertension mainly applies the blood pressure detection method. The categories of blood pressure are classified according to the blood pressure value. It is known that cuff-type blood pressure detection is often affected by the white-coat phenomenon, reading problems, and difficult operation for the elderly, etc. [42]. These factors limit its widespread application. However, hypertension and other cardiovascular diseases are becoming increasingly serious in the world, with an urgent demand for new blood pressure detection and hypertension assessment methods [43]. PPG signals can reflect the state of the cardiovascular circulatory system in real time, and the measurement method is noninvasive. These factors, as well as the PPG’s convenience and low cost, have allowed it to become an important means of noninvasive detection and assessment of cardiovascular health [44]. However, since PPG signals are very susceptible to the patient’s age, movement, respiration, and other interferences, previous research based on the PPG signal has often encountered difficulty in accurately identifying and extracting feature points. The development of deep learning technology provides a potential way around these difficulties by automatically and robustly extracting the features from raw signals. 

This paper presented an automatic classification and risk stratification method for hypertension using PPG signals based on a continuous wavelet transform and pretrained convolutional neural network (GoogLeNet). This method has many potential applications, such as in ICU wards and wearable PPG devices (e.g., finger probes or smartwatches, etc.). It should be noted that for a wearable device, the trained deep learning model will need to be extensive. Indeed, a wearable device always has low computational complexity and small memory. However, cloud computing technology has become very mature. Wearable devices are used to collect signals, and cloud platforms with trained deep learning models are used to predict real-time data and send results to a wearable device. These techniques have been applied in many commercial smart devices.

In the development and evolution of hypertension, human blood pressure is experienced at different levels, including normotension, prehypertension, hypertension, and other stages. For the early diagnosis and assessment of hypertension, the timely detection of prehypertension and the accurate diagnosis of hypertension are significant factors. Therefore, in this study, three hypertension classification trials were set: NT versus PHT, NT versus HT, and non-HT (NT plus PHT) versus HT. Table 1 demonstrates the classification performance of the three classification trials. As can be seen from Table 1, all three F1 scores were more than 0.8, with those over 0.9 classified as hypertension. As this study was based on the PPG signals of patients in ICU wards, it was accepted that the quality of the PPG signal would not be ideal. Therefore, these results show the potential outcomes of applying this method. 

PAT is a highly studied parameter in new types of cuffless blood pressure detection methods and has been explored by many studies in the context of blood pressure detection [14,45]. In a previous study, blood pressure classification using PAT and PPG features that were extracted from ECG and PPG signals was also conducted [24]. The same dataset used in that study was used in this study. From Table 1, we can see that the F1 score of this study was higher than the classifier using 10 PPG features. This means that GoogLeNet is easily able to achieve better performance than extracting PPG morphological features based on the poor quality PPG signals that are collected from elderly ICU patients. In addition, this study achieved performance similar to artery wave propagation theory. This means that this study, which only used raw PPG signals, has a strong potential to replace the ECG and PPG method to classify hypertension. It is established that for artery wave propagation theory, it is necessary to collect ECG and PPG signals simultaneously and to accurately identify and extract the morphological features of these signals. This requirement greatly limits its wider application. Compared to traditional research methods, the proposed method has more potential applications and greater practical value. The advantages are more obvious. 

Exploring novel blood pressure detection and hypertension risk stratification was the main focus of this paper, and the authors have researched this area extensively in the past [23,24,46]. It is evident that difficulty arises mainly from the extraction of features from physiological signals with differing qualities. Therefore, a new exploration using deep learning technology was conducted. The results demonstrated good performance and potential. Deep learning techniques have grown quickly in recent years. For example, ECG classification was first studied using a deep learning model in 2016 [47,48,49]. It provided a new approach to processing and analyzing ECG signals. Blood pressure is a basic and important physiological parameter that is challenging to detect and classify using non-invasive PPG signals. Deep learning techniques have an advantage when used for the classification of new blood pressure categories. The current study helps us to understand how deep learning may be used for the assessment of blood pressure. With the development of deep learning in recent years, more complete deep learning toolboxes have been released, especially MATLAB R2018, introduced in the methodology section.

A bigger dataset is always ideal for such studies. For our study, we used signals collected from 121 subjects, and each recording was 120 s in length per subject. When creating the training and testing sets, each subject signal was cut down to 24 five-second windows. Therefore, 2904 signal segments were used to create the RGB images. The training set consisted of 2323 images, and the testing set consisted of 581 images. For these thousands of images, the training time of each trial lasted more than 350 min. The computation complexity was high and, therefore, taken into consideration during the training phase.

It is noteworthy that many studies have used the MIMIC database assuming that all the simultaneously collected signals were synchronized [50,51,52,53,54]. However, the creators of the MIMIC database have reported errors in the data matching and alignment in of some recordings, as mentioned by Clifford et al. [14], confirming that not all the signals were synchronized. This issue is not important for us, as we analyzed the PPG signal alone.

Although the method proposed in this study has outstanding advantages, its challenges cannot be ignored. The limitations of this study were that the dataset used was still small, and each sample was only 2 min in length. Much more validation and optimization with more clinical data will continue in future work. In addition, a main problem of this study was that the training stage was too long. However, this is a common problem in the training of deep learning models. Another time-consuming stage was the continuous wavelet transform and conversion to the scalogram. However, the scalogram was beneficial in showing the different frequency components of the PPG signal, which can improve the classification performance. Fortunately, the computational complexity problem can be ignored because, in practice, only a fully trained model is used, which requires a short time for prediction. The specific advantages and disadvantages are summarized as follows:

Advantages:

1. Can be completely automated
2. No need to extract morphological features
3. No special requirement for the signal quality of the PPG signal
4. Applicable to real-time processing of big data

Disadvantages:

1. Demands higher processing power and resources
2. Needs more training time
3. Requires training with large-scale data

The ECG and PPG methods are indeed very promising methods for noninvasive continuous blood pressure monitoring. The PAT method has a relatively stable blood pressure correlation, which has also been studied and confirmed by previous research. ECG and PPG can provide a more clinical diagnosis with ECG signals, in addition to providing blood pressure detection. However, it is known that it is not easy to obtain a stable ECG signal using an ECG device and a PPG device in different settings. Additionally, the acquisition of ECG signals is a barrier to the wider application of this technology. It is also known that, despite the ease of operating an oscillographic electronic sphygmomanometer, many people are unable to use it properly. Therefore, reducing the complexity of the installation and operation is still an issue that has to be considered. This study showed that using deep learning technology and raw PPG signals is very promising and forms a novel exploration for deep learning potential in blood pressure assessment.

This study’s proposed method could play a major role in the early detection of hypertension in low- and middle-income countries (LMICs). With the use of only PPG signals, which are easy to use, simple, and effective, utilizing the six-step framework for a global health approach [55] can lead to decreased morbidity and mortality rates in LMICs.

## 5. Conclusions

In clinical settings, the computer-aided diagnosis of hypertension can significantly reduce the workload of doctors and nurses, especially in Intensive Care Unit wards. An automatic diagnosis and warning system for hypertension is, therefore, a necessary component of timely and accurate screening and treatment of the disease. We hypothesized that deep learning will provide better results when compared to the classical method. Results showed that the proposed deep learning method (combining continuous wavelet transform and the GoogLeNet) achieved higher accuracy than PPG features extracted from PPG signals. The proposed deep learning method does require a higher quality PPG signals and does not require extraction of the PPG morphological feature, making it easy to apply in many situations. In future research, using an increased sample size, we will be able to continue improving the performance for hypertension risk stratification. This paper used a non-GPU device. In the future, we plan to further expand the dataset, include more noise sample records and use a GPU-based workstation for model optimization and learning.

## Figures and Tables

**Table 1:** Classification performance of the proposed deep learning method and feature-based methods on the same recordings from the MIMIC database [33]. Note, NT, PHT, and HT represent normotension, prehypertension, and hypertension, respectively. PAT stands for pulse arrival time, CWT stands for continuous wavelet transform, KNN stands for k-nearest neighbors.

	Trial	Feature	Classifier	F1
This study	NT (46) vs. PHT (41)	CWT scalogram	GoogLeNet	80.52%
	NT (46) vs. PHT (34)	CWT scalogram		92.55%
	(NT + PHT) (87) vs. HT (34)	CWT scalogram		82.95%
PAT feature [24] (ECG and PPG signals)	NT (46) vs. PHT (41)	PAT and 10 PPG features		84.34%
	NT (46) vs. HT (34)	PAT and 10 PPG features	KNN	94.84%
	(NT+PHT) (87) vs. HT (34)	PAT and 10 PPG features		88.49%
PPG features [24] (only PPG signal)	NT (46) vs. PHT (41)	10 PPG features		78.62%
	NT (46) vs. HT (34)	10 PPG features	KNN	86.94%
	(NT+PHT) (87) vs. HT (34)	10 PPG features		78.44%
